# Association of common medical comorbidities with early renal damage in the Chinese tropics with essential hypertension

**DOI:** 10.1186/s12882-021-02576-8

**Published:** 2021-11-05

**Authors:** Yuzhuo Zhang, Ying Zhao, Chenglu Wei, Yongrong Li, Hira Aslam, Qingmin Feng, Qing Huang, Yu Zheng, Feifen Lv, Wei Hao, Jike Li

**Affiliations:** 1 First Affiliated Hospital of Hainan Medical University, Haikou, China; 2 Marketing Department, Sanofi (Hangzhou), Haikou, China; 3 Cardiovascular Department, Xi’an Hospital of Traditional Chinese Medicine, No.69, Fengcheng 8th Road, Weiyang District, Xi’an City, 710021 Shaanxi Province China

**Keywords:** Chinese tropics, Early renal damage, Essential hypertension, Urine albumin/creatinine ratio

## Abstract

**Background:**

Urine albumin/creatinine ratio (UACR) is an important marker of early renal damage (ERD) caused by hypertension. Recent studies showed that blood pressure was a significant inverse association with temperature and climate. The purposes of our study were sought to explore the association of common medical comorbidities with ERD, and find independent risk factors to ERD in Chinese tropics with essential hypertension.

**Methods:**

From January 2018 to December 2019, we assessed UACR in a total of 599 hypertensive Chinese Hainan patients. We defined ERD as a UACR between 30 mg/g and 300 mg/g. We analysed differences between qualitative variables using the chi-squared (χ^2^) test. We calculated correlations between UACR and age, hypertension duration (HD), systolic blood pressure (SBP), and diastolic blood pressure (DBP) using the Spearman’s rho test. To determine the odds ratio (OR), we evaluated binary logistic regression models.

**Results:**

Among the 599 patients, 281 (46.9%) were found to have ERD. ERD and factors related to sex, body mass index (BMI), and SBP did not differ significantly (all, p>0.05). Our main findings showed that age, HD, and DBP were associated with ERD (p<0.01, respectively). Furthermore, age ≥ 65 years, HD ≥10 years, DBP ≥ 90 mmHg, SBP ≥ 160 mmHg, and diabetes differed significantly according to ERD status (*p* < 0.05, respectively). In multivariate analysis using stepwise regression, age (OR = 1.468), DBP (OR = 1.853), and diabetes (OR = 2.031) were significant independent predictors of ERD. The area under the receiver operating characteristic (ROC) curve was 0.677, and the sensitivity and specificity of the optimal cut-off value were 44.5 and 81.1%, respectively.

**Conclusions:**

Common medical comorbidities are associated with ERD; age, DBP, and diabetes are independent risk factors for ERD in patients with essential hypertension who live in the Chinese tropics. Early monitoring of the UACR, as well as control of blood glucose and DBP, can effectively delay ERD.

## Background

Hypertension is a risk factor for cardiovascular disease and chronic kidney disease (CKD) [1]. According to the China Hypertension Survey (2012–2015), 23.2% (≈244.5 million) of the Chinese adult population (≥18 years) had hypertension during that time period; it is the leading modifiable risk factor for coronary heart disease (CHD) and represents the top cause of death in China [2]. Hypertensive nephropathy is the second leading cause of CKD [3]. However, if early renal damage (ERD) is intervened in as early as possible, the renal function of patients with hypertension will obtain better protection. The indexes of ERD include homocysteine, β2-microglobulin, cystatin-C, serum creatinine, angiotensin-II, microalbuminuria, and urine albumin/creatinine ratio (UACR), yet among those indexes, the level of UACR is relatively high reproducible for clinical practice, and less influenced by urine volume, time, or diet value [4].

For a long time, uncontrolled hypertension could lead to renal failure, a marker of ERD through UACR testing [5, 6]. Moreover, the UACR, measured in a spot urine sample, is highly correlated with 24-h urine albumin excretion as a predictor of the development and progression of diabetic and non-diabetic renal diseases, as well as of incident hypertension and cardiovascular mortality [7–9]. UACR is little affected [4], while several aspects such as temperature and climate remain uncertain and await elicitation. Currently, hypertensive patients in North China have higher levels of blood pressure than those in the south [10]; one possible cause may be seasonal variation. Multiple studies have indicated that seasonal variation could affect hypertension in Western or Chinese populations [11–13]. Hainan island is located in the southernmost part of the country, where the annual average temperature ranges from 22.5 ~ 25.6 °C, and there is non-significant temperature variation in the tropical, oceanic climate. Previous studies on hypertension have mainly focused on mainland China; data on China’s tropical islands related to the detection and control of hypertension are absent. Thus, we aimed to explore the association of common medical comorbidities with ERD, and to identify independent risk factors for ERD with essential hypertension in the Chinese tropics, eventually providing new data for local public health authorities.

## Methods

### Study design and participants

From January 2018 to December 2019, we selected 599 hypertensive patients at both outpatient and inpatient department based on inclusion and exclusion criteria in the First/Second Affiliated Hospital of Hainan Medical University. We included patients if they had newly diagnosed hypertension by measured systolic blood pressure (SBP) ≥ 140 mmHg and/or diastolic blood pressure (DBP) ≥ 90 mmHg three times on different days, or had suffered from hypertension and were taking blood pressure medication. We excluded patients who took high doses of vitamin C in the previous 10 h; engaged in intense exercise in the previous 24 h period; stood for a prolonged period over 6 h; had a fever or infection (especially in the urinary system); were menstruating or pregnant; had a history of primary/secondary renal disease, blood system diseases, malignant tumours, connective tissue disease, severe hepatic insufficiency, hyperthyroidism, or chronic heart failure; or had significant proteinuria (>150 m/24 h), which can be induced by all kinds of diseases.

### Hypertension diagnosis

All patients who met the diagnostic criteria for hypertension had an SBP ≥ 140 mmHg or a DBP ≥ 90 mmHg in the sitting position after 5 min of rest, as determined by the general practitioner in triplicate; we also included those taking antihypertensive drugs.

### UACR monitor

**Fig. 1:**
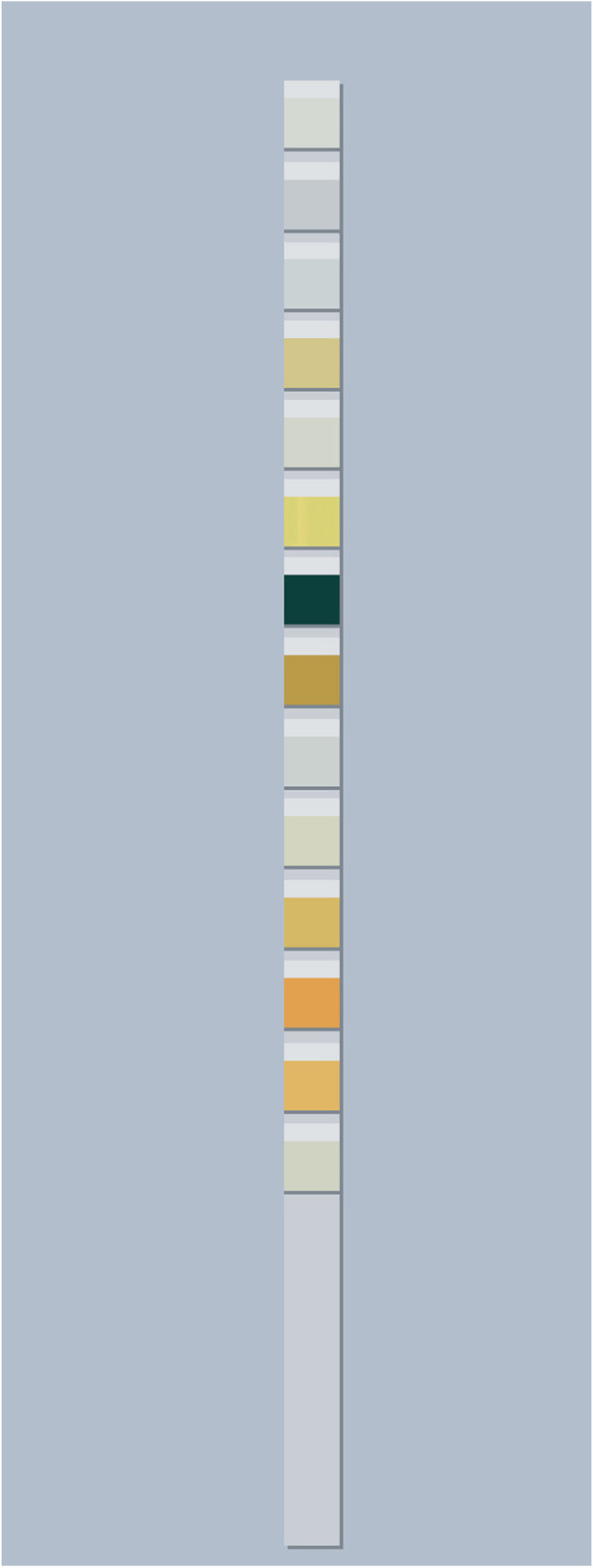
Clinitek strips were used to measure urine albumin concentration and urinary creatinine

**Fig. 2:**
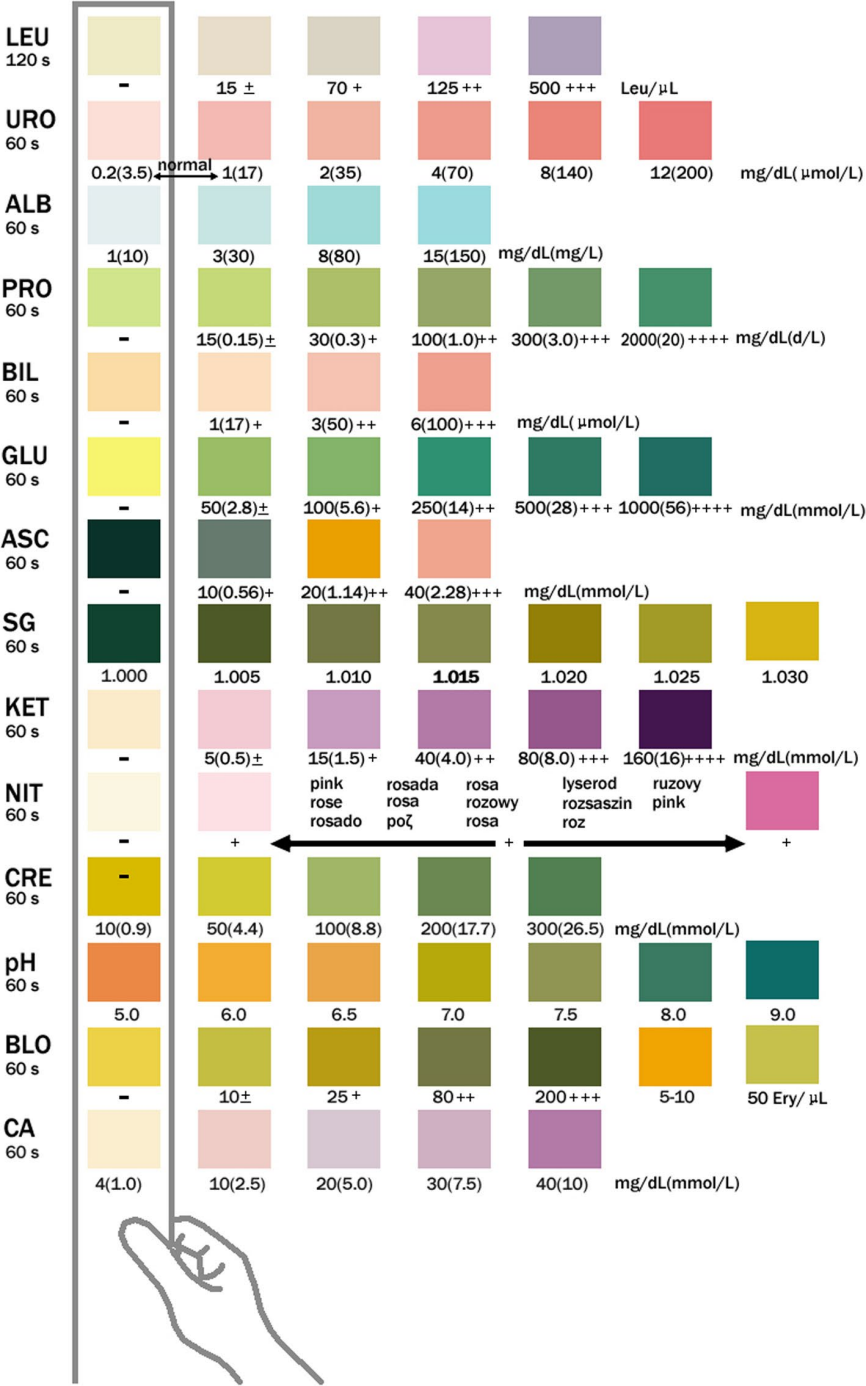
The Clinitek strips were developed to stand for 60 s. We read the values of microalbuminuria and creatinine using a visual method to make a comparison with the chromatogram. Abbreviations: MAU, microalbuminuria; LEU, leukocyte; URO, urobilinogen; ALB, albumin; PRO, protein; BIL, bilirubin; GLU, glucose; ASC, ascorbic acid; SG, specific gravity; KET, ketone; NIT, nitrite; CRE, creatinine; BLO, blood; CA, calcium

**Fig. 3:**
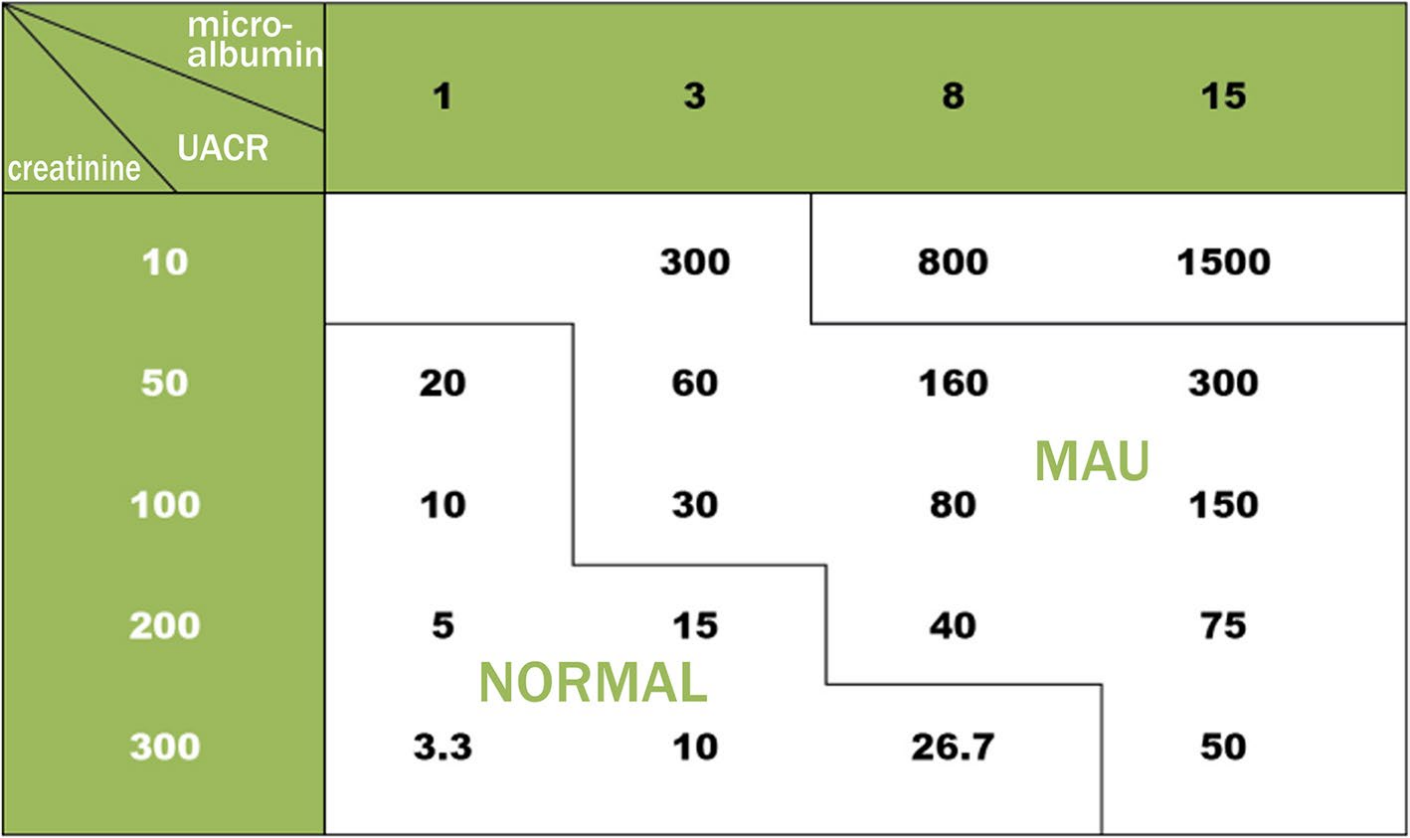
According to the test paper, the UACR value is found in the UACR reading metre. Note: the unit of microalbuminuria and urinary creatinine is mg/dl, and the unit of UACR is mg/g. Abbreviations: UACR, urine albumin/creatinine ratio

To monitor the UACR, all urine specimens were supplied in the morning. We gauged urine albumin concentration and urinary creatinine with Clinitek Strips (dry chemistry assay, ACON Biotech, China). The colour appeared after letting the Clinitek Strips stand for 60 s, and we read the values of microalbuminuria and creatinine via a visual method as compared to the chromatogram (Figs. 1 and 2). Finally, according to the interpretation of the test paper, we found the corresponding UACR value to be in the UACR reading metre (Fig. 3). We divided all subjects into two groups: no ERD (*n* = 318) with a UACR of 0–29 mg/g, and ERD (*n* = 281) with a UACR of 30–300 mg/g.

### Covariates

We measured body mass index (BMI), daytime SBP, and daytime DBP. We recorded sex, age, hypertension duration (HD), diabetes, CHD, and stroke. We diagnosed diabetes using medical history if the patient was under antidiabetic treatment, or based on two or more fasting or 2-h post-meal plasma glucose determinations of ≥7.0 mmol/L (126 mg/dL) and ≥ 11.1 mmol/L (200 mg/dL), respectively. BMI < 24 is represented as the normal range.

### Statistical analysis

Continuous variables are presented as the means ± standard deviations (SD); categorical variables are shown as percentages. We performed statistical analysis of quantitative variables using an independent t-test, and we analysed the differences between qualitative variables via the chi-squared (χ^2^) test. Since the UACR is an ordinal scale, we calculated correlations between UACR and various factors (age, HD, SBP, DBP) with Spearman’s rho test. To determine the odds ratio (OR) of ERD, we evaluated binary logistic regression models. We performed a binary logistic regression analysis using SPSS software, version 19.0 (Copyright 1989–2010 Inc., IBM company, IL, USA; ibm.com/cn-zh/analytics/spss-statistics). We considered *p* < 0.05 to be statistically significant. The OR indicates the ERD risk of subjects with higher values compared to those with lower values; 95% confidence intervals (CIs) were reported in the multivariate logistic regression models. For the detailed analysis process, we referenced the work of Lavanderos [14]. To further verify the optimal cut-off value, we calculated it for ERD using the receiver operating characteristic (ROC) curve methodology, described elsewhere [15].

## Results

### Basic characteristics

Of all 599 hypertensive patients, 281 (47%) had ERD. There were no differences in male sex, BMI, SBP, or antihypertensive drug (*p* > 0.05). Clearly, the age of onset was higher in the ERD group than in the non-ERD group (61.239 ± 11.706 vs. 64.081 ± 12.352, *p* = 0.004), and the same as in the DBP (81.940 ± 11.888 vs. 85.348 ± 13.660, *p* = 0.001). Moreover, the ERD had a longer HD (6.372 ± 4.352 vs. 7.650 ± 5.630, *p* = 0.002) (Table 1).

**Table 1:** Baseline characteristics of patients with essential hypertension in the Chinese tropics

Parameter	No ERD (*n* = 318)	ERD (*n* = 281)	*P*
Age (y)	61.239 ± 11.706	64.081 ± 12.352	0.004*
Male sex (%)	152/318 (47.799%)	123/281 (43.772%)	0.328
BMI (kg/m2)	24.026 ± 2.827	24.433 ± 2.800	0.087
HD (y)	6.372 ± 4.352	7.650 ± 5.630	0.002*
SBP (mmHg)	138.623 ± 19.584	141.473 ± 22.540	0.098
DBP (mmHg)	81.940 ± 11.888	85.348 ± 13.660	0.001*
Antihypertensive drug	1.450 ± 0.657	1.505 ± 0.677	0.308

Continuous variables are presented as the means ± standard deviations (SD); categorical variables are shown as percentages

*Abbreviations*: *ERD* Early renal damage, *BMI* Body mass index, *HD* Hypertension duration, *SBP* Systolic blood pressure, *DBP* Diastolic blood pressure.

*p < 0.05 vs no ERD

### Correlation analysis

**Fig. 4:**
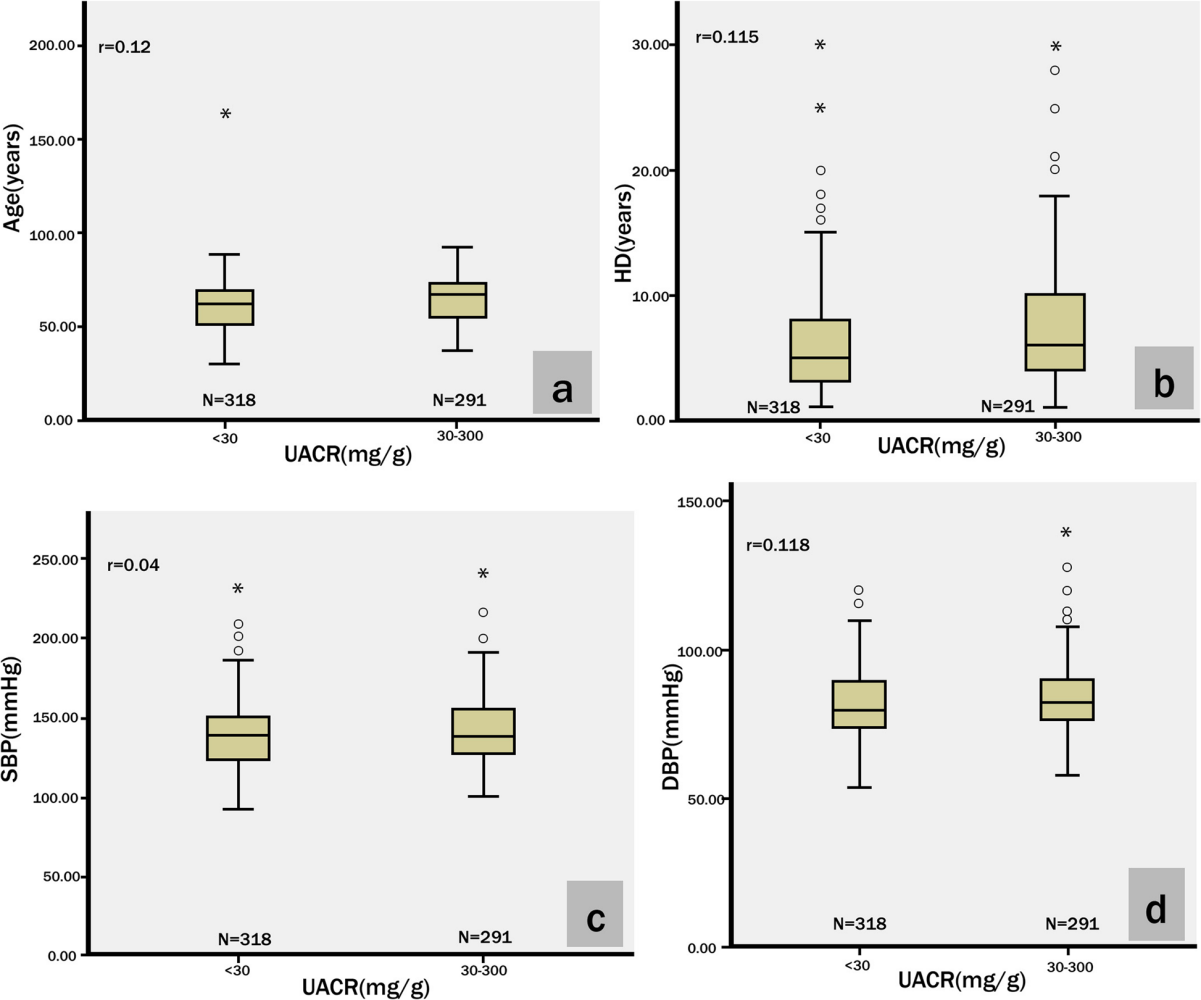
Spearman’s Rho test was used to analyse correlations between age, HD, SBP, DBP and UACR, and *p* < 0.05 to be statistically significant. Abbreviations: HD, hypertension duration; SBP, systolic blood pressure; DBP, diastolic blood pressure; UACR, urine albumin/creatinine ratio

We used Spearman’s rho test to analyse correlations between ERD and related variables (Fig. 4). There were significant Spearman correlations between ERD and age (*r* = 0.12, *p* = 0.003), HD (*r* = 0.115, *p* = 0.005), and DBP (*r* = 0.118, *p* = 0.004). The Spearman correlation between ERD and SBP was not significant.

### Single factor analysis

To screen for significant, independent, qualitative variables—including sex, age, BMI, HD, DBP, SBP, and combined disease (CHD, diabetes, stroke)—we employed the χ^2^ test. Compared with no ERD, ERD was associated with age (< 65 y vs. ≥65 y, χ^2^ = 17.064, *p* < 0.001), HD (< 10 y vs. ≥10 y, χ^2^ = 4.441, *p* = 0.035), DBP (< 90 mmHg vs. ≥ 90 mmHg, χ^2^ = 5.521, *p* = 0.019), SBP (<160 mmHg vs. ≥ 160 mmHg, χ^2^ = 4.412, *p* = 0.036) and diabetes status (χ^2^ = 7.229, *p* = 0.007), which were significantly different according to the presence of ERD. There was no significant difference between ERD and sex, BMI, CHD, or stroke (*p* > 0.05) (Table 2).

**Table 2:** The single factor analysis of χ^2^ test was used on ERD

Factors	Parameter	No ERD (*n* = 318)	ERD (*n* = 281)	x^2^	*P*
Sex	Male	152 (55.3%)	123 (44.7%)		
	Female	166 (51.2%)	158 (48.8%)	3.477	0.323
Age (y)	<55	96 (57.8%)	70 (42.2%)		
	≥55	222 (51.3%)	211 (48.7%)	2.074	0.150
	<65	194 (61.0%)	124 (39.0%)		
	≥65	124 (44.1%)	157 (55.9%)	17.064	<0.001*
BMI (kg/m2)	<24	159 (58.0%)	115 (42.0%)		
	≥24	147 (50.3%)	145 (49.7%)	3.363	0.067
HD (y)	<10	248 (55.6%)	198 (44.4%)		
	≥10	70 (45.8%)	83 (54.2%)	4.441	0.035*
DBP (mmHg)	<90	231 (56.3%)	179 (43.7%)		
	≥90	87 (46.0%)	102 (54.0%)	5.521	0.019*
SBP (mmHg)	<140	161 (51.1%)	154 (48.9%)		
	≥140	157 (55.3%)	127 (44.7%)	1.043	0.307
	<160	273 (55.0%)	223 (45%)		
	≥160	45 (43.7%)	58 (56.3%)	4.412	0.036*
Diabetes	Yes	54 (42.5%)	73 (57.7%)		
	No	264 (55.9%)	208 (44.1%)	7.229	0.007*
CHD	Yes	72 (55.4%)	58 (44.6%)		
	No	246 (52.5%)	223 (47.5%)	0.351	0.553
Stroke	Yes	57 (51.4%)	54 (48.6%)		
	No	261 (53.5%)	227 (46.5%)	0.165	0.685

The single factor analysis of χ^2^ test

*Abbreviations*: *ERD* Early renal damage, *BMI* Body mass index, *HD* Hypertension duration, *DBP* Diastolic blood pressure, *SBP* Systolic blood pressure, *CHD* Coronary heart disease

* *p* < 0.05

### Binary logistic regression model analysis

To evaluate the weight of the dependent variable and to correct the interaction effect among the above variables, we used a binary logistic regression model for analysis. As outlined in Table 3, age, DBP, and diabetes had statistically significant results for predictors of ERD, since the risks of ERD included age (< 65 y vs. ≥ 65 y, OR = 1.468), DBP (< 90 mmHg vs. ≥ 90 mmHg, OR = 1.853) and diabetes (OR = 2.031). Although HD and SBP were significantly different in the χ^2^ test, they could not become independent predictors of ERD with binary logistic regression model analysis. Therefore, age, DBP, and diabetes were independent risk factors for essential hypertension in patients who suffered from ERD.

**Table 3:** Results of binary logistic regression model analysis for predictors of ERD

Risk Factors	Odds Ratio	95% CI	*P*
Age (y)	1.468	1.179–1.829	0.001*
HD (y)	1.230	0.963–1.570	0.098
DBP (mmHg)	1.853	1.418–2.516	<0.001*
SBP (mmHg)	0.625	0.365–1.068	0.086
Diabetes	2.031	1.334–3.092	0.001*

We performed a binary logistic regression analysis using SPSS software

*Abbreviations*: *ERD* Early renal damage, *HD* Hypertension duration, *DBP* Diastolic blood pressure, *SBP* Systolic blood pressure

*p < 0.05

### Defining cut-off values

**Fig. 5:**
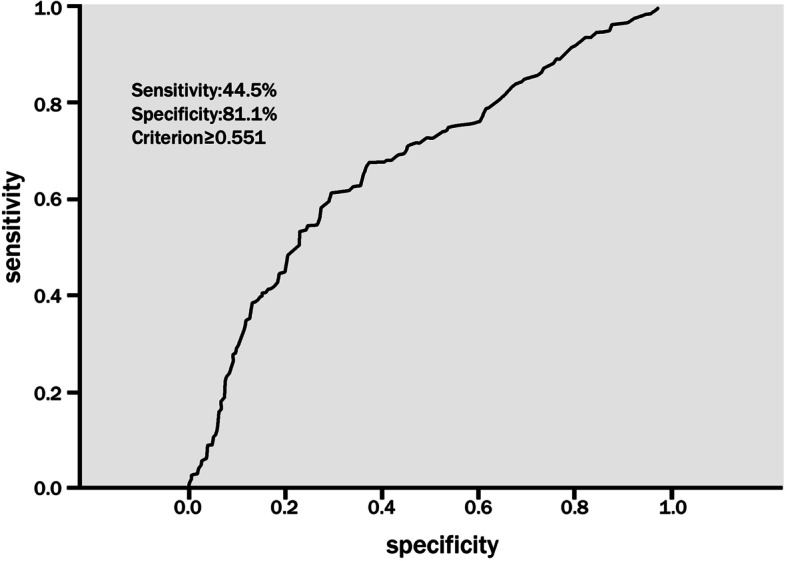
ROC curve for the prediction of ERD through age, DBP and diabetes mellitus. Note: The area under the ROC curve was 0.677 (95% CI: 0.633–0.720, p<0.001). The sensitivity and specificity of the optimal cut-off value were 44.5 and 81.1%, respectively. Abbreviations: ROC, receiver operating characteristic; ERD, early renal damage; DBP, diastolic blood pressure

We employed ROC curves to analyse the relationship between ERD and risk factors (age, DBP, and diabetes). The area under the ROC curve was 0.677 (95% CI: 0.633–0.720, *p* < 0.001). We chose the optimal cut-off value of ≥0.551; the sensitivity and specificity of the optimal cut-off value were 44.5 and 81.1%, respectively (Fig. 5).

## Discussion

Hypertensive renal damage caused by uncontrolled blood pressure alters renal structure and function, and is the second most important cause of CKD after diabetes [16], which seriously threatens patients’ health and lives. Therefore, the identification and intervention of ERD are urgently needed to delay the progression of CKD. UACR has been used to identify sensitive indexes of ERD that reflect not only glomerular basement membrane injury but also vascular endothelial structure and dysfunction; these are associated with morbidity and mortality [17]. Measurement of UACR is recommended in all hypertensive patients (ESC Classes of recommendations: IB) [18]. Because of its feasibility and ease of operation, the UACR measurement is widely used in the clinical diagnosis and monitoring of ERD. Controlling hypertension effectively is helpful in reducing UACR levels, which is an extremely important method to slow down the progression of ERD caused by hypertension.

We initially observed the relationship between UACR and ERD in patients with hypertension living in the tropics of China. In our study, 46.7% were from the outpatient department. To ensure the consistency of blood pressure measurement and UACR detection time, we gauged daytime blood pressure. Our research demonstrated that DBP over 90 mmHg is an independent risk factor for predictors of ERD. Our results indicated that high DBP is associated with increased cardiovascular risk and is more commonly elevated in younger individuals (< 65 years). The Framingham heart study signalled that DBP tends to decline from midlife as a consequence of arterial stiffening [19], and in middle-aged and older people, increased pulse pressure (the difference between SBP and DBP values) has additional adverse prognostic significance [20]. DBP decreased by 10 mmHg or less than 75 mmHg is associated with the reduction of age-related stroke and ischemic heart disease [21]. DBP reflects the magnitude of peripheral vascular resistance, which is affected by heart rate and the elasticity of the arteriole wall, and is related to the compliance of arteries; moreover, strengthening peripheral arterial vascular resistance leads to an increase in DBP [22]. Target organ injury caused by SBP mainly consists of left ventricular hypertrophy and enlargement, while systemic arteriolar lesions are primarily caused by an increased wall/cavity ratio and reduced lumen diameter, resulting in ischaemia of target organs such as the heart, brain, and kidneys [22]. Therefore, the effect of DBP on renal injury is more notable. Patients with early hypertension and renal injury should more strictly control DBP and reduce peripheral resistance to effectively delay the progression of renal vascular injury. However, there is also the undeniable fact that SBP is closely tied to ERD. Our findings revealed that high SBP (≥ 160 mmHg) differs significantly from ERD. Hence, we speculate that higher SBP may be associated with ERD defined by increased UACR of 30 to 300 mg/g. However, the levels of DBP and SBP have received much attention, and are significantly correlated with the degree of microalbuminuria [23]. In addition, DBP may better reflect peripheral resistance than mean SBP, as it was steadier than SBP when the subjects engaged in daily activities. Different age stages will make a large difference between DBP and SBP, just as DBP may better reflect peripheral resistance in relatively young people, while SBP may be associated with a predominance of arterial stiffness in old age [24].

The latest research has shown that there is a significant inverse association between BP and temperature; tighter BP control is more necessary in winter than in summer, especially in a colder climate [25]. The China Kadoorie Biobank study [25] verified that blood pressure varies seasonally, and each 10 °C lower ambient temperature is associated with a 6.9/2.9 mmHg higher SBP/DBP. Su et al. demonstrated that the detection and control of hypertension are down/up respectively when the temperature rises [26]. The HOMED-BP study suggested that the large-variation group (which increases significantly from winter to summer; systolic/diastolic ≥9.1/≥ 4.5 mmHg) was confirmatory for major adverse cardiovascular events [27]. Anti-dipper hypertension is the chief hypertensive type in aged individuals; the management of nocturnal BP is especially critical. Notwithstanding, Hainan’s warm, humid climate makes the area have little influence on night hypertension, and the tropical marine climate has an all-weather suitable temperature, with little significant change between day and night. Further, a light diet, sweat, and excessive salt excretion in Hainan may reduce ERD caused by hypertension. Currently, Chinese hypertension standard centres have been opened in many hospitals to reduce hypertensive target organ damage, which is a particularly important measure of Chinese chronic disease management. We found that advanced age (≥ 65 years) is an independent risk factor for ERD. Ageing is an irreversible risk factor, and most elderly people adopt more sedentary lifestyles. A lack of exercise leads to weight gain. Thus, the prevalence of hypertension worldwide will continue to rise [28]. Advanced age should be given more attention regarding the stable control of blood pressure and the protection of target organs. A previous study implied that in patients aged < 60 years, DBP provides significant value for predicting a morbid cardiovascular event [29]. Hence, how to delay senility and maintain the function of viscera remains a hot topic of research. Our results also signalled that control of DBP < 90 mmHg could be used for risk stratification of hypertensive patients without clinically significant renal damage. Recent evidence hints that UACR is a predictor of the development and progression of diabetes mellitus and cardiovascular disease [7, 9]. We also found that UACR is highly correlated with diabetes. However, our results indicated that both CHD and stroke have no significant differences compared to UACR. We think the reasons are that the favourable marine climate and a low salt and fat diet with seafood lead to delays in the progression of both diseases. Simultaneously, owing to Chinese chronic disease insurance, almost all patients with stage 3 hypertension can receive effective antihypertensive treatment. Above all, early monitoring of the index UACR, as well as control of blood glucose and DBP, can effectively delay EDR in patients with essential hypertension in the Chinese tropics.

### Limitations

Anti-hypertensive drugs were not covered in this article.

## Conclusions

Our findings imply that common medical comorbidities are associated with ERD in patients with essential hypertension in the Chinese tropics. Age, DBP, and diabetes are independent risk factors of ERD. Early monitoring of UACR, as well as control of blood glucose and DBP, can effectively delay EDR in patients with essential hypertension in the Chinese tropics.

## Data Availability

The datasets generated and/or analyzed during the current study are available from the corresponding author.

## Abbreviations

UACR: Urine albumin/creatinine ratio
ERD: Early renal damage
HD: Hypertension duration
SBP: Systolic blood pressure
DBP: Diastolic blood pressure
OR: Odds ratio
CKD: Chronic kidney disease
CHD: Coronary heart disease
LEU: Leukocyte
URO: Urobilinogen
ALB: Albumin
PRO: Protein
BIL: Bilirubin
GLU: Glucose
ASC: Ascorbic acid
SG: Specific gravity
KET: Ketone
NIT: Nitrite
CRE: Creatinine
BLO: Blood
CA: Calcium
BMI: Body mass index
CI: Confidence interval
ROC: Receiver operating characteristic
